# Animal-assisted therapy at a University Centre for Palliative Medicine – a qualitative content analysis of patient records

**DOI:** 10.1186/s12904-017-0230-z

**Published:** 2017-10-02

**Authors:** Andrea Schmitz, Melanie Beermann, Colin R. MacKenzie, Katharina Fetz, Christian Schulz-Quach

**Affiliations:** 1 Interdisciplinary Centre for Palliative Medicine, Heinrich Heine University Hospital Dusseldorf, Dusseldorf, Germany; 2 LVR Clinic of Psychiatry, Psychosomatic and Psychotherapy for children and adolescence, Viersen, Germany; 3 Maudsley Training Programme, Institute of Psychiatry, Psychology and Neuroscience, King’s College London, London, UK; 4 St. Christopher’s Hospice, Sydenham, London, UK; 5 Institute of Medical Microbiology and Hospital Hygiene, University Hospital, Heinrich Heine University Dusseldorf, Dusseldorf, Germany; 6 Chair of Research Methodology and Statistics in Psychology, Department of Psychology & Psychotherapy, Faculty of Health, Witten/Herdecke University, Witten, Germany

**Keywords:** Palliative care, Animal-assisted therapy, Dog

## Abstract

**Background:**

Animal-assisted therapy (AAT) is a therapeutic concept, which has only recently been explored in more detail within the palliative care setting. A programme of AAT was begun in June 2014 at the Interdisciplinary Centre for Palliative Medicine of the University Hospital Dusseldorf, Germany. The AAT sessions were performed by two trained and certified dog assistant therapy teams (DATT). To date only very limited scientific data are available with regard to feasibility, therapeutic indications and efficacy of AAT in palliative care. The present qualitative study aims to describe the first year’s practice and experience of AAT after implementation as an integral part of adjunctive therapy options offered within an academic palliative care centre.

**Methods:**

This study is a qualitative content analysis of all post-encounter protocols of AAT interventions recorded by the dog handlers from June 2014 through May 2015. Qualitative content analysis was conducted according to Mayring’s approach; the report followed the recommendations of the Standards for Reporting Qualitative Research (SRQR).

**Results:**

Fifty-two patients received 84 AAT interventions, with only 18 patients receiving more than one intervention due to discharge or death. In 19 cases relatives also participated in the AAT session. The inductive coding process yielded four main categories. One hundred and fifty-three codes related to the content and structure of the AAT sessions, with physical contact with the dog taking considerable precedence. The AAT sessions included conversations with the dog handler, 10.5% of which related to the current health state as well as to discussions around death and dying. Eighty-nine codes related to perceived emotional responses, with pleasure being the most often observed response. Two hundred and seventeen codes related to the effects of the AAT sessions, identifying the dog as a catalyst of communication and observing patients’ physical activation or relaxation.

**Conclusions:**

AAT may constitute a valuable and practicable adjunct to the interdisciplinary therapeutic repertoire of palliative care in the hospital setting. The results of this study suggest that patients may potentially benefit from AAT in terms of facilitated communication, positive emotional responses, enhanced physical relaxation or motivation for physical activation. These early stage results will need to be followed-up by more robust study designs.

## Background

Palliative care attends to health care needs of persons with progressive and life-limiting diseases. Common symptoms are pain, anxiety and psychosocial distress, which is why animal-assisted therapy (AAT) may constitute a valuable therapeutic approach for the benefit of palliative care patients, but little is known about this approach so far.

The WHO definition of palliative care (2002) emphasises improving the quality of life of patients and their families and the interprofessionality and comprehensiveness of this therapeutic approach [1]. One main objective of palliative care is to relieve or alleviate patients’ symptom burden to the best possible extent. Also, relatives and friends of patients are often in a state of existential distress and in need of professional support. The use of AAT in palliative care is a relatively recent and not yet routinely established therapeutic concept, even though both share the goal of improving patients’ quality of life [1, 2].

The International Association of Human-Animal Interaction Organizations (IAHAIO) (2014) defines AAT as follows*: “Animal Assisted Therapy is a goal oriented, planned and structured therapeutic intervention directed and/or delivered by health, education and human service professionals. Intervention progress is measured and included in professional documentation. AAT is delivered and/or directed by a formally trained (with active licensure, degree or equivalent) professional with expertise within the scope of the professionals’ practice. AAT focuses on enhancing physical, cognitive, behavioural and/or socio-emotional functioning of the particular human recipient.*”[3].

Humans are innately social beings; they need social relationships and emotional bonds. This need is not necessarily restricted to relationships between humans but can cross the species boundary, therefore enabling bonding between humans and animals. Wilson describes this kind of bond – called *biophilia* – as a product of evolutionary development, proposing that humans have an urge to affiliate with other forms of life [4].

The knowledge that animals improve the well-being of humans is far from new and the development of its deliberate therapeutic application dates back to the late eighteenth century. Florence Nightingale, for instance, mentioned in her *Notes on Nursing* that „a small pet is often an excellent companion for the sick, for long chronic cases especially „[5]. Boris Levinson described the beneficial effect of his dog’s presence during therapeutic interactions with his patients [6]. Today a broad range of scientific findings suggest a beneficial effect for humans as a direct result of interacting with an animal (human-animal interaction, HAI) [7]. For an example, Vernooij and Schneider refer in their analysis of HAI to psychoanalytic theory and conceptualise the function of the animal as: object for identification, projection and motivation, which can also serve as a transition object and catalyst in processing difficult emotions [8]. The most common type of AAT-related HAI involves dogs [2].

Beetz et al. showed in their review about psychophysiological effects of human-animal interactions well-documented benefits for stress-related parameters such as decrease in cortisol plasma levels, heart rate, and blood pressure and some limited evidence for reduction in epinephrine and norepinephrine plasma levels. The authors discuss activation of the oxytocin system as the underlying key mechanism [9]. Current therapeutic indications of AAT for medical purposes are based on these scientific findings showing, for instance, reduced fear and anxiety after an AAT intervention in psychiatric patients, especially in cases of situational fear prior to medical procedures [10].

Currently, there is a dearth of research examining the application of AAT in palliative care, especially regarding psychological aspects. Engelman et al. described in their anecdotal study 2013 that AAT “can be an effective method for reducing pain in palliative care patients” [11]. Engelman described a 51-year-old patient in a palliative care setting, who as a result of low mood and anger progressively withdrew socially and asked to be “left alone”, however, he engaged in AAT and after only one session re-engaged with his environment [11]. With regard to the behavioural functioning, Berry showed that AAT might improve behavioural activation in geriatric patients [12]. Most of the available literature, however, is based on anecdotal research, opinion pieces, and poorly designed studies. The critical review by Chur-Hansen 2013 therefore concluded that currently “there is a weak evidence base for AAT (...) in palliative care” [13]. Consequently, formulating clear therapeutic indications and therapy objectives for AAT in palliative care, especially for improvement of psychological well-being, is not possible at present and research is needed.

The present qualitative study aims to describe the first year’s practice and experience of AAT after implementation as an integral part of adjunctive therapy options offered by an academic palliative care centre.

## Methods

In the present study, the Standards for Reporting Qualitative Research (SRQR) were adopted for the presentation of our data [14].

Based on the limited evidence available, the Interdisciplinary Centre for Palliative Medicine (ICP) of the University Hospital Dusseldorf, Germany, has defined psychologically distressing symptoms as suitable therapeutic indications of AAT (see Table 1). The indication is determined by the attending physician and the psycho-oncologist or psychotherapist.

**Table 1:** Psychological Indications of AAT at the Interdisciplinary Centre for Palliative Medicine (ICP) of the University Hospital Dusseldorf, Germany

Indications of AAT	
Severe tension	
Adjustment disorder	
Depression	
Demoralisation syndrome	
Terminal delirium	
Anxiety and fear	

The study design is based on the retrospective analysis of dog handlers‘ protocols of AAT sessions. An inductive approach was used following Mayring’s model of qualitative content analysis [15]. The aim was to generalise from single phenomena.

### Research team and reflexivity

The evaluation of research data was conducted by AS, the (former) medical chief, who implemented AAT at the ICP in 2014, and principal researcher of the ICP Dusseldorf, Germany, and MB as a doctoral student. They analysed the data after it was de-identified. CSQ, the (former) deputy medical chief and principal researcher of the ICP Dusseldorf, Germany, examined and reviewed the research results. To take possible role-conflicts, conflicts of interest or bias into account, all three were neither present during the AAT sessions, nor during the dog handlers’ documentation of the sessions. No instructions beyond the standard clinical governance rules were given to the dog handlers as to how to document the AAT session. The dog handlers’ notes were not discussed with them afterwards. These precautions were put in place to clearly separate between clinical intervention and the related research. The SRQR guideline was used for quality assurance purposes within this research. During data analysis, researchers maintained reflexivity by regular research meetings of all investigators, discussions about coding rules and the developing category system, as well as using a reflexive journal for methodological decisions and documenting means of conflict resolution for divergent understandings of data.

### Setting

This study was conducted at the ICP of the Heinrich Heine University Hospital Dusseldorf, Germany. The interprofessional team of the ICP Dusseldorf, Germany, attends to the needs of more than 600 patients and their relatives per year. At the ICP Dusseldorf, patients are treated on the palliative care ward as well as on general wards by means of a palliative consultation service team. At the 8-beds specialized palliative care unit patients at the end of life are mainly diagnosed with end-stage cancer, chronic organ failure and/or neurological diseases. Patients have a varying degree of symptom burden needing treatment on the physical, psychological, social and spiritual level. Approximately 40% of inpatients get discharged from the unit after treatment, whilst 60% die during their admission [16]. The average length of stay is 12 days [17].

### Intervention

All AAT interventions were performed in the palliative care unit. All documented AAT sessions between June 1st, 2014 and May 31st, 2015 were included. During this time, two dog assisted therapy teams (DATT) performed AAT at the ICP Dusseldorf, Germany. AAT was only offered to patients without known allergies or aversion to animals who were suffering from psychological distress (see Table 1).

Each AAT therapy session followed a clear structure, which was nevertheless adapted to individual patient needs and wishes. It consisted of four stages – introduction, observation, contact and farewell (see Table 2). During each session the therapist applied three main strategies: free interaction (e.g. playing with the dog), directed interaction (e.g. observation task) and ritualised interaction (e.g. signal response) [8].

**Table 2:** AAT structure according to Gottschling [18] as applied at ICP Dusseldorf

Schedule	Content
Introduction	Greeting of patient
Observation	Introducing the dog and motivational conversation, during which the patient has the opportunity to observe the dog (predominantly directed interaction)
Contact	Patient-dog activities (e.g. stroking, giving treats, games, physical activity), communication with dog handler about the dog and other topics of interest to the patient (predominantly free interaction)
Farewell	Farewell ritual, arranging for another therapy session, etc. (ritualised interaction)

All forms of interactions could be used in the stages of observation and contact. Generally, free interaction becomes increasingly relevant with increasing numbers of sessions.

One of the trained and certified DATT consisted of a therapist with background in social work and a therapy assistant dog. The other team consisted of a therapist with background in education and a therapy assistant dog. Both were trained and certified to national standards, one of them to ESAAT standards. The research material consisted of the protocols written by the therapists after each AAT session.

### Analysis

Analyses were performed using Word, MAXQDA 11 and Excel (Microsoft Office 2011). Demographic data were analysed and registered using Excel (Microsoft Office 2011). AAT session protocols were extracted from individual electronic patient files. We collected demographic data (gender, age), medical (diagnosis, hospital stays) and organizational data (duration of AAT intervention). Data were analysed only after sufficient anonymisation. With regard to verbatim excerpts, we used pseudonyms and we took particular care to eliminate any personally identifiable information. Documented AAT sessions were added to a software program, MAXQDA 11, and analysed according to Mayring’s model of qualitative content analysis [15]. We did not paraphrase our material, since the AAT protocols were already short and condensed. We conducted an open, inductive analysis. After familiarisation with the raw data a coding scheme was developed in a multi-level process. First MB and AS performed the coding process independently, then any discrepancies were discussed and new codes or code definitions were created. To enhance the trustworthiness and credibility of our data analysis, triangulation was conducted with CSQ.

After confirmation of sufficient inter-coder reliability, the text passages were then subsumed to formulate categories. Parallel data for 19.2% were coded, with an inter-coder reliability of 88% (Cohen Kappa 0.82).

Ethical approval was granted by the Ethics Committee of the Medical Faculty of Heinrich Heine University Dusseldorf, Germany protocol number 5105, 2015/06/01.

## Results

Between June 1st, 2014 and May 31st, 2015, 52 patients received AAT at the ICP Dusseldorf, Germany.

### Patient characteristics

**Fig. 1:**
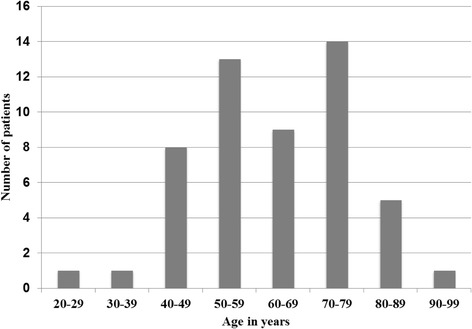
Age distribution among the patients who received AAT (*n* = 52)

Of the 52 patients receiving AAT, 32 were female (61%) and 20 were male (39%). Median age was 65 years (mean 63.3; 28–90 years; see Fig. 1). Forty-nine patients were treated on the palliative care ward and three patients were treated on general wards by the ICP palliative consultation service. All patients suffered from a progressive terminal primary disease and had been referred for palliative care. Forty-seven patients had an oncological disease and five patients a non-oncological disease, i.e. cerebral apoplexy (*n* = 2), terminal heart failure (n = 2), terminal renal failure (*n* = 1).

Seventeen patients had a documented history of companion animal ownership, of which 14 owned dogs. A further 17 patients had a documented history of experiences with dogs, but without companion animal ownership. For 21 patients, the dog handlers’ protocols included comments regarding pre-existing symptom burden during AAT session. The most frequently symptoms mentioned were exhaustion/fatigue, followed by pain and dyspnoea. Twelve patients showed visible signs of active pre-existing symptom burden during their sessions, but all patients wished to continue their respective AAT sessions.

### AAT characteristics

**Fig. 2:**
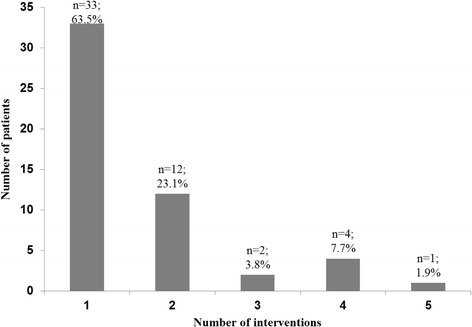
Number of interventions, 52 patients received a total of 83 AAT interventions

During the 12-month observation period, 52 patients received 84 AAT sessions by two teams of therapy companion dogs and their respective handlers (per patient: median 1.0, mean 1.6; see Fig. 2). Among those, most of them received a single intervention. Only 18 patients had a second AAT session even though the dog handlers’ protocols showed 38 patients requesting further AAT sessions.

**Fig. 3:**
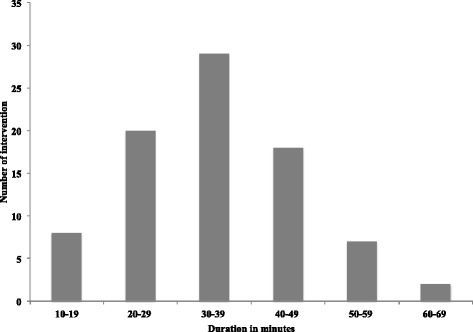
Duration of AAT intervention

With regard to AAT duration, the median was 30 min (mean 32.7 min; 10–67 min; see Fig. 3).

### Categories

The defined raw material produced 544 codes yielding 21 subcategories. Inductive coding produced four main categories:

1. ***AAT practice and environmental factors***
2. ***Content of AAT sessions***
3. ***Effect of AAT***
4. ***Behavioural activation through AAT***

The respective subcategories reflect the wide range of the main categories. The coding guide in Table 3 provides a summarising overview of the codes. Table 4 presents a list of the most important codes.

**Table 3:** Coding guideline

Category	Code	Subcode	Code definition	Anchor example	Coding rule
AAT practice / environmental factors	Intervention site	Patientroom; bed / wheelchair / couch	All coding items describing the patient room; bed / wheelchair / couch as the site of the intervention	“Patient is in her room, lying in her bed” (P44)	
		Garden / outdoors; bed / wheelchair / walking	All coding items describing the garden / anywhere outdoors as the site of the intervention and / or the patient is sitting in a wheelchair or walks freely	“Leads her outside himself with the wheelchair” (P35)	
		Multifunction room; wheelchair / Bed	All coding items describing the multifunction room as the site of the intervention; degree of mobility is mentioned: wheelchair or bed	“Patient is accompanied by physician and comes into the multifunction room to meet Lotti.” (P39)	
	Integration of relatives / relatives	Active participation in AAT	Coding items related to relatives receiving AAT and participating actively	“Relative, i.e. husband is offered a session with therapy dog Lotti. Was pleased, suggested a walk on the hospital premises, talked about his situation when arriving at the hospital, on the ward. Declined offer to walk dog on the leash but was intensively aware of the dog’s repeatedly initiated physical contact and stroked and touched Lotti at regular and short intervals for a short time, later on continually and always accepted her prompts to touch and stroke her.” (P16)	
		Active participation in AAT patient + relatives	All coding items mentioning the presence of relatives and their active participation in or integration into the AAT session	“Mother and daughter then try out a snack-game together. Both enjoy it and try various things” (P36)	
		Passive participation in AAT patient + relatives	All coding items mentioning the presence of relatives, albeit in the background and without actively participating in the AAT session	“Sister barely participates in the interaction” (P42) “Wife keeps to the background, upon request handed dog to husband, became more active only when Lotti was sitting on a chair beside the husband’s bed.” (P49)	
		Passive participation in AAT	Coding items related to relatives receiving AAT without the patient and staying passive	“Son stays in the background does not act, only watches.” (P30)	
	Discontinuation of intervention	General	Coding items mentioning the discontinuation of the AAT session	“Discontinuation of AAT session.” (P12)	
		Criterion	Coding items related to the reason for discontinuation of AAT session	“However, describes being too weak, too tired, it is too much for her. Discontinuation upon patient’s request” (P40)	Including problems during AAT session (“No further feeding or licking of patient’s left hand because of an inflammation of index finger, in order to protect patient and dog.”)
	Second contact	not desired	Coding items indicating no desire for another AAT session	“Patient refuses dog therapy. Reacts negatively, wishes to be left alone/ in peace.”	
				(P41)	
		Desired but not performed due to hospital discharge / deterioration in health and functional status / death	Coding items mentioning patient’s desire for another AAT session which was, however, not performed. Therefore no documentation; Follow-up whether patient was discharged, lacking adequate health / functional status for an AAT session, had died.	“Patient sets a goal of going for a walk with Lotti, she would be pleased about a further visit.” (P50)	
		Desired and performed	Coding items related to another AAT session being carried out	“Second contact with patient. Patients is pleased to see therapy dog again.” (P2)	
Content of AAT sessions	Performed AAT intervention	Exercises with therapy dog	All coding items related to exercises / activities during the AAT session	“Bottle trick” (P23)	Including feeding the dog
		Positioning of therapy dog	All coding items mentioning the positioning of the therapy dog	“Quedo is layed down by her side and initiates contact by licking” (P11)	
		Patient-centred session	All coding items indicating that the AAT session is tailored to current patient needs	“Patient tells about her day so far and that the drugs have made her tired and that she therefore does not wish for “big action” with Lotti, but that she is pleased to see her. Lotti greets her at the bedside, initiates physical contact by laying her head onto the bed and nudging the patient. Patient is already talking about a photo session planned for next week. Then she shows her own photos and the video made during the last contact.” (P18)	E.g. changing from active to restful activities because patient / relative signals exhaustion.
		Photo	All coding items related to a picture / video being taken during the AAT session	“Right from the start it is very important for him to take a picture” (P25)	
		Adressing death / dying	Coding items indicating death / dying as a conversation topic during the AAT session	“Only speaks once and shortly about the approaching end” (P33)	
		Adressing disease and hospital stay	Coding items mentioning the patient’s disease and/or related hospital stays / therapeutic measures / limitations in everyday life	“Talks about her family, her staying here, also about her disease and the effects on her being” (P52)	Including comments on general state of health, independently of AAT session
		Stroking of dog	All coding items mentioning initiated / maintained tactile contact with the dog	“He strokes Quedo and grasps his fur” (P1)	
Effect of AAT	Effect of AAT	Closeness / intimacy and trust	Coding items mentioning closeness, intimacy, trust, bond	“She calls the dog to her, enjoys the closeness with her, strokes her only shortly” (P51)	
		Calmness / relaxation	All coding items related to relaxation / deceleration of life speed / calmness; either mentioned by the patient or perceived by the dog handler	“She seems relaxed and calm again” (P45)	
		Self-efficacy	Coding items indicating actions of the patient and the ensuing effects; patient realization that his/her actions have effect on environment	“And realizes she can bring about things” (P44)	
		Distraction	Coding items mentioning the patient’s distraction because of the AAT session	“Is happy ‘about the distraction from this disease’” (P52)	
		Catalyst for communication	Coding items related to AAT facilitating and initiating communication, providing conversation topics, serving as ice-breaker	“He speaks about his experiences with dogs and animals, how beneficial these were for him, occasionally about the relationships one has with animals. This causes him to think about his children, he talks about them, his suicide attempt and his other thoughts.” (P32)	Patient - dog handler patient – dog patient – relatives relatives- dog relatives - dog handler
		Rejection / aversion	Coding items describing rejection of / aversion to AAT sessions or parts of the intervention	“She stroked her body, emphasised now that she did not like the dog licking her and lead Lotti together with me in a way which allowed her to reach her back and to stroke her there.” (P16)	
		Activation	Coding items describing that the AAT session has an activating effect on the patient / relative	“He seems content, activated, a bit excited.” (P20)	
		General effect of contact with animals	Coding items describing the effects of animals on human beings, what they evoke in human beings, what they can mean for human beings	“He speaks about his experiences with dogs and animals, how beneficial these were for him, occasionally about the relationships one has with animals.” (P32)	
		Emotions / observer perception	All coding items related to the dog handler perceiving and identifying emotions of the patient or relative	“She is very pleased, almost seems to be touched” (P44)	Including tears interpreted as sadness / joy, interpreted by dog handler; facial expression unambiguous / clearly understandable
		Emotions / self-perception	All coding items related to the patient / relative identifying his/her emotions or speaking about emotions	“During that she talks in detail about her husband’s dogs, the associated feelings, e.g. pride, but also fear and grief” (P36)	Including relatives’ comments on behalf of the patient
		emotions / humour	Coding items related to patient currently being able so show humour by means of comment, laughing, joking	“Made some jokes, laughed several times a bit cautiously – but sincerely about her thoughts.” (P51)	
		Emotions / understanding / reflexion	Coding items related to the patient perceiving sent emotions, reflecting on them, interpreting them and/ or reacting to them	“She watches and peers at Lotti intensively, verbally mirrors her behaviour and declares her being motivated, adopts this motivation for herself.” (P52)	
Patient’s behavioural activation through AAT	Behavioural activation	Yes; own ideas for exercises	All coding items identifying motivated behaviour during the interaction with the dog and patient’s own ideas for exercises are expressed and / or executed	“Patient asks for dog-snacks and bottle himself, he decides himself and becomes active.” (P38)	
		Yes; instruction / suggestions for exercises necessary	All coding items related to the patient being motivated but in need of ideas and suggestions on how to interact with the dog	“He needs a bit of prompting to initiate contact with Lotti” (P47)	
		No motivation	All coding items related to the patient being listless or not motivated. Nevertheless, mention of patient having and expressing ideas for exercises is possible	“Patient does not respond to suggested activities” (P12)

**Table 4:** Coding list

Category	Subcategory	Exemplifying citations
AAT practice and environmental factors	*Intervention site*	▪ “Comes into the multifunction room, is sitting in a wheelchair.” (P27)
	*Discontinuation of intervention*	▪ “Patient discontinues the AAT session due to pain. Patient requests another appointment.” (P10)
	*Second contact*	▪ “She would like another visit on Thursday “if she is still there”.” (P36)
	*Integration of relatives*	▪ “While playing with their grand-father who provides instruction and acts as an “expert” for Lotti, they try out numerous things and are impressed by what “Grandpa” is able to do and what he already knows after only 2 meetings with Lotti.” (P25)
Content of AAT session	*Stroking of dog*	▪ “Patient was relaxed, permitted manual guidance, stroked Lotti like that, felt dog’s heartbeat and breathing, stroking with manual guidance.” (P20)
	*Exercises with therapy dog*	▪ “She gave visual signals like Sit and Give Paw, rewarded the dog with treats from a spoon while commanding Lotti to wait or to come to her and acted with self-assurance.” (P16)
	*Positioning of the dog*	▪ “Lotti is led to the bedside. I take the patient’s hand and forearm to extend it towards the dog, there is skin contact and his fingers stroke the fur.” (P30)
	*Patient centred session*	▪ “After greeting her and a bit of caressing the dog I ask her indirectly if stroking the dog is enough for her or whether she wants to get a bit more active with the dog. Upon which she asks which material we used the last time.” (P46)
		▪ “Right from the start it is very important for him to take a photo.” (P25)
	*Addressing disease, dying, death\disease and hospital stay*	▪ “Talks about illness, about humans and animals, wishes related to animals, emotions evoked by them. Starts to swallow hard when talking about last diagnosis, eyes full of tears, then concentrates on Lotti and is able to enjoy some moments with her in the bed.” (P44)
Effect of AAT	*Emotions*	▪ “She verbally reflected on Lotti’s behaviour, made some jokes, laughed several times a bit cautiously - but sincerely - about her thoughts.” (P51) ▪ “Observes her closely while trying to analyse whether she is tired or if she wants to go outside. I mirror Lotti’s behaviour for her and show her that she is actually completely relaxed and that Lotti adapts to her mood.” (P51)
	*Catalyst for communication*	▪ “Lotti is a facilitator for communication.” (P28) ▪ “Patient opens up emotionally during the conversation.” (P5)
	*Activation*	▪ “Patient speaks of tiredness but becomes more active again during the ball game.” (P13)
	*Relaxation*	▪ “She seems relaxed and calm again. Says Lotti is looking for her calmness and makes her calm.” (P45) ▪ “Appears visibly weaker and tired to me; Lotti repeatedly lies down in front of her bed, too, adopts her calmness; Patient realises that also and is able to accept it.” (P18)
	*Self-efficacy*	▪ “She seems to feel a connection to Lotti, realizes that she can make her come to her, even control her through hand commands like Sit, Down, Give Paw, and realizes the effects of her own actions.” (P46)
	*Intimacy and trust*	▪ “He notes being aware of the fact that his manner makes Lotti accept this kind of trust.” (P42) ▪ “He becks Lotti to him, purposefully seeking to establish physical contact. He strokes and cuddles her very intensively. He wants her to lie in his bed. Lotti lies down next to him and allows every degree of closeness he is seeking.” (P42)
	*Distraction*	▪ “Towards the end, she speaks, for the first time, about her disease, her thoughts about dying, her life, her values. At the same time, her voice becomes weaker. But then she turns to Lotti again, is able to find pleasure in the interaction and decides herself to try a trick as a farewell.” (P36)
	*Aversion*	▪ “Patient refuses dog therapy. Reacts negatively, wishes to be left alone/ in peace.” (P41)
	*General effects of contact with animals*	▪ “He speaks about his experiences with dogs and animals, how beneficial these were for him, occasionally about the relationships one has with animals. This causes him to think about his children, he talks about them, his suicide attempt and his other thoughts.” (P32)
Behavioural activation through AAT	*Motivation available*	▪ “Adopts this motivation for herself.” (P52)
	*No motivation*	▪ “But shows no motivation or ideas to become active herself.” (P46)

#### AAT practice and environmental factors

For the most part, AAT sessions took place *in the patient’s room*, sometimes also *in the garden/outdoors* or *in a multipurpose intervention room* on the ward. On some occasions AAT continued whilst patients were transferring from one environment to another (e.g., from the garden to their room). In 15 patients AAT was *discontinued*; criteria of discontinuation: pain, tiredness, loss of concentration and anxiety of a to close bond to the dog. Eighteen patients received more than one AAT intervention session. For a further 20 patients, protocols indicated expressed desire for further sessions. The median interval between repeated AAT sessions was five days. In 19 cases relatives participated in the patients’ respective AAT sessions or separate AAT session were offered for those relatives.

#### Content of AAT sessions

One hundred and fifty-three codings related to the content, i.e. the structure and activities of the AAT sessions. *Stroking the therapy dog* was described most often.

*“(…)permitted manual guidance, stroked Lotti like that, felt dog’s heartbeat and breathing, stroking with manual prompt” (P20).*

Protocols included typical *exercises with the therapy dog*, chiefly feeding the dog as well as exercises and activities requiring a certain measure of physical energy and coordination.

*“Giving the dog a snack and opening of snack-box with help. Sit-gesture with right hand for Sit and Down.”* *(P34).*

*“(…) we used the stroll outside for an “exercise session” for Lotti – walking the dog with wheelchair.”* *(P37).*

During interventions the dog could *lie on the bed*.

*“(…)Having the dog put her head on his arm, he can feel the pressure of its weight. Taking snacks out of his left hand, from under his arm, repeated nudging, skin contact, feeling and touching of fur.” (P30).*

The content and activities of an AAT intervention could be adapted to the current needs and inclination of the respective patient. This *patient-centred implementation* was documented in 12 patients.

*“After greeting her and a bit of caressing the dog I ask her indirectly if stroking the dog is enough for her or whether she wants to get a bit more active with the dog. Upon which she asks which material we used the last time. I unpack some items and she starts to associate them with interactions and memories”* *(P46).*

Upon request or permission by the patient, the AAT session was captured in a picture.

*“(…) right from the start it is very important for him to take a picture.” (P25).*

The therapeutic interaction promoted conversation between dog handlers and patients. In the case of 16 patients, protocols included conversation topics involving patient’s current health state, with disease-related fatigue being foremost. Five patients talked about *dying and death*, three of those found distraction in the interactions with the therapy dog.

*“Talks about illness, about humans and animals, wishes related to animals, emotions evoked by them. Starts to swallow hard when talking about last diagnosis, eyes full of tears, then concentrates on Lotti and is able to enjoy some moments with her in the bed..”* *(P44).*

#### Effect of AAT

In order to investigate the effect of AAT on palliative care patients, we determined codes identifying the effect of AAT. All in all, 217 codes could be assigned to nine subcategories. The dog handlers recorded patient *emotions* evoked during AAT, chiefly *pleasure*.

The dog handlers’ *observer perception* produced 80 codes related to *emotions*, headed by *pleasure* (*n* = 33) and *self-satisfaction* (*n* = 7). Further emotions evoked during AAT and observed by the dog handler or relatives were: *sadness, fear*. Furthermore, it was remarkable to note in nine patients that the AAT intervention gave rise to episodes involving *humour*.

*“She verbally reflected on Lotti’s behaviour, made some jokes, laughed several times a bit cautiously – but sincerely – about her thoughts.”* *(P51).*

A therapy dog uses its behaviour, i.e. its body language, to send signals. Some patients realised and understood this and reflected on it. Thus, observing the therapy dog and verbalising the mirrored behaviour enabled these patients to reflect upon their own emotions.

*“She watches and peers at Lotti intensively, verbally mirrors her behaviour and declares her being motivated, adopts this motivation for herself.”* *(P52).*

AAT can serve as a *catalyst for communication* (*n* = 30).

*“Today he accepts considerably more attempts at conversation; whereas I talked a lot during the initial contact because of his many questions, today he is the one to talk the most. He speaks about his experiences with dogs and animals, how beneficial these were for him, occasionally about the relationships one has with animals. This causes him to think about his children, he talks about them, his suicide attempt and his other thoughts.”* *(P32).*

As a consequence of the targeted interaction with the therapy dog, patients feel either activated or *relaxed* and *calm*. Another finding is patients’ developing *self-efficacy* (*n* = 20) as a result of the interactions with the therapy dog.

*“She seems to feel a connection to Lotti, realizes that she can make her come to her, even control her through hand commands like Sit, Down, Give Paw, and realizes the effects of her own actions.”* *(P46).*

The physical contact and the interaction with the dog may promote a sense of *closeness* and *trust*. AAT stimulates patients’ *distraction* by shifting their attention on the dog.

*“(…)is happy ‘about the distraction from this disease”. (P52).*

In some cases, however, the AAT interventions resulted in patients’ *rejection* due to a too quickly evolving situation of closeness or a general aversion to AAT.

*“She does not wish to be visited by Lotti again because she is afraid of becoming too involved, “Better not let it become too close”, “Better slowly reduce it, who knows”; but at the end she states, “Maybe we'll see each other again sometime, somewhere.””* *(P51).*

*“Patient refuses to feed treats but has difficulties explaining that he does not like it. Reacts negatively, wishes to be left alone / in peace.” (P41).*

Patients described the effect of the animals on them as restorative, beneficial, enriching and positively emotional (*n* = 5).

*“When we said good-bye he once more talked about his own pets, switching to the therapy dog and the energy that animals give you and how they seek contact with human beings by themselves.”* *(P16).*

#### Behavioural activation through AAT

During AAT a varying degree of behavioural activation was observed. All in all, the coding process yielded 34 codes relating to dog handlers’ documentation regarding patients’ behavioural activation. Protocols included information on whether a patient could be motivated by means of the interaction with the therapy dog, whether they needed support in interacting or dealing with the dog or whether they could not be activated or motivated during the AAT session in general.

*“Adopts this motivation for herself.*” *(P52).*

*“But shows no motivation or ideas to become active herself.” (P46).*

## Discussion

The present qualitative study described the first year’s practice and experience of AAT in inpatient care at an academic palliative care centre. Only a third of the patients had a second AAT session. For those who did not receive a further intervention, the following two reasons were identified: first, hospital discharge before the next planned AAT session; secondly, patient death. The frequent occurrence of patient death in palliative care is a common phenomenon which constitutes a limiting factor and a challenge for rigorous evidence-based palliative care research [19].

The present study described the current practice of a newly implemented AAT concept with AAT sessions currently offered twice a week, however, most patients in our study received only one AAT session. Relatively short average length of stay at the specialised palliative care unit serves as a limiting factor for repeated interventions, however, this study was not aimed at identifying the optimal dose of AAT for a diverse range of indications and this will need to be looked at in research projects going forward. Interestingly, there is no robust evidence at present that higher frequency of AAT interventions improves outcomes. To the contrary, a study by Banks and Banks looked at reducing loneliness in an elderly population in long-term care facilities by AAT, comparing AAT facilitation once a week and three times a week, respectively. They found that AAT once a week was as effective as three times a week in reducing loneliness in long-term care residents [20].

AAT offered by the ICP Dusseldorf, Germany, is tailored to individual patient needs. During the 12-month observational period, the average duration of an AAT session was 30 min. In some patients, the AAT session was discontinued ahead of schedule due to patients’ symptom burden like exhaustion and fatigue. However, most of these patients expressed their desire to continue with the AAT session in spite of existing symptom burden. This finding supports the positive effect of tailoring therapy duration to individual patient needs. At the same time it is important to also consider the welfare of the therapy dog, for instance in terms of signs of discomfort and exhaustion. It is for this reason that the International Society for Animal Assisted Therapy (ISAAT) and the European Society for Animal Assisted Therapy (ESAAT) have published AAT quality criteria and guidelines to ensure the wellbeing of the involved animals [21].

Even though there are various palliative care facilities currently offering AAT or animal-assisted activity (AAA), there is still a paucity of scientific research demonstrating their efficacy and feasibility [22]. A German study conducted by Gottschling et al. on the efficacy of AAT in palliative care patients suggests that the targeted use of therapy dogs may significantly improve patients’ well-being [23].

To date, there exist no clearly formulated therapeutic indications of using AAT in palliative care settings. Wohlfahrt and Olbrich state that merely a general objective statement is loosely based on the ICD list or the ICF model [21].

It is for this reason that the researchers of the present study chose patients based on psycho-socio-emotional burden and distress and therefore with regard to possible treatment objectives of AAT, e.g. improved communication, increased emotional stability, patient motivation and activation, improved relaxation and development of self-satisfaction.

### Effects of AAT sessions

AAT sessions consist of four stages (see Table 2). The contact phase is mainly intended for those activities targeting the respective therapy objectives.

In this context, tactile interaction with the therapy dog was of central importance. It was achieved by means of touching, patting or stroking the therapy dog or having it lie on the patient bed beside the patient. In those interactions dog handlers’ observation records frequently identified observing patients to become more relaxed. Several studies documented that human relaxation as a consequence of interaction with animals was closely associated with increased levels of oxytocin, endorphins and a decreased cortisol level [9, 24, 25]. As a more intermediate effect, and different from the immediate relaxation, AAT seemed to cause behavioural activation in some palliative care patients. Petting the dog and building rapport appeared to motivate patients to initiate reciprocity and willingness to engage in playful interaction with the dog. This observation has been reported in the literature before. For an example, Berry described the same finding for AAT as utilised in working with geriatric patients [12].

What is more, we found that dog handlers’ protocols often mentioned AAT-induced emotions. Pleasure was recorded most often and was associated with the presence of and interaction with the dog. On the other hand, they occasionally observed sadness during the encounters with the therapy dog, but also recorded that the therapy dog eventually calmed and distracted the patients during the AAT session. Another emotion observed in the patients by the dog handlers was humour. Penson et al. postulated that humour, used with sensitivity and adequately, may constitute a valuable addition to health providers’ therapeutic repertoire [26]. For several palliative care patients, the dog handlers documented observing increased self-efficacy as a result of AAT. This finding is in line with the study by Berget et al., who reported that AAT with farm animals may have a positive effect on patients’ self-efficacy and coping ability [27].

The protocols often included reports of how patients frequently talked about topics like their own disease, death and dying and that the encounter with the therapy dog enhanced the initiation of such conversations, supporting the assumption that AAT may be a valuable therapeutic tool to promote positive social interaction and communication. Lang et al., who investigated the effect of AAT in reducing anxiety in acute schizophrenic patients, reported that a reduction of anxiety may promote initiation of interpersonal contact and communication [28].

The present study described a single centre application of AAT and gave a first descriptive indication of potential beneficial effects of AAT in a palliative care setting. These findings can serve as a basis for more research exploring AAT in palliative care as an adjunctive therapeutic approach to reduce patients’ symptom burden with specific emphasis on ameliorating psychosocial symptoms.

### Limitations

This descriptive, qualitative study has clear limitations and caution should be used in generalising from these single centre findings. The small study sample, the low number of therapy sessions and the qualitative analysis of AAT session protocols written by two different dog handlers constitute clear limitations of the present study. Moreover, the findings are based on the perceptions of the handlers and are not necessarily based on what actually occurred. In addition, there was a considerable variance in the dog handler’s open text protocols, which was due to the insufficient standardisation of documentation and differing professional backgrounds of the dog handlers.

## Conclusions

This study describes the first year’s practice and experience of AAT after implementation into an academic palliative care centre within a specialized inpatient unit. We described the structure and process of implementing AAT as an adjunctive therapy option and described potentially beneficial patient outcomes on a variety of psychosocial distress symptoms as observed in this particular cohort of palliative care patients.

There is an urgent need of further qualitative research studies to thoroughly investigate possible effects on palliative care patients. It is further recommended to develop clearly formulated and research-based therapeutic indications of AAT in palliative care. It might also be of scientific interest to analyse videotaped patient-animal encounters to be able to describe non-verbal interaction phenomena in detail.

## Abbreviations

AAA: Animal-assisted activity
AAT: Animal-assisted therapy
ESAAT: European Society for Animal Assisted Therapy
HAI: Human-animal interaction
IAHAIO: International Association of Human-Animal Interaction Organizations
ICP: Interdisciplinary Center for Palliative Care
ISAAT: International Society for Animal Assisted Therapy
SRQR: Standards for reporting qualitative research
